# Lung Density Measurement in Fontan Patients Using Zero Echo Time Sequences

**DOI:** 10.1007/s00246-025-03825-5

**Published:** 2025-03-18

**Authors:** Alessia Callegari, Konstantinos Zeimpekis, Barbara E. U. Burkhardt, Emanuela R. Valsangiacomo Buechel, Fraser M. Callaghan, Jakob Usemann, Martin Hersberger, Christian J. Kellenberger, Julia Geiger

**Affiliations:** 1 Pediatric Heart Center, University Children’s Hospital Zurich, Zurich, Switzerland; 2 Children´s Research Center, University Children’s Hospital Zurich, Zurich, Switzerland; 3 Department of Nuclear Medicine, Inselspital, Bern University Hospital, University of Bern, Bern, Switzerland; 4 Center for MR-Research, University Children’s Hospital Zurich, Zurich, Switzerland; 5 Department of Respiratory Medicine, University Children’s Hospital Zurich, Zurich, Switzerland; 6 Division of Clinical Chemistry and Biochemistry, University Children’s Hospital Zurich, University of Zurich, Zurich, Switzerland; 7 Department of Diagnostic Imaging, University Children’s Hospital Zurich, Zurich, Switzerland; 8 Pediatric Heart Centre, Division of Pediatric Cardiology, University Children’s Hospital Zurich, Lenggstrasse 30, CH-8008 Zurich, Switzerland

**Keywords:** Magnetic resonance imaging, Fontan circulation, Single ventricle, Lung density, Biomarkers

## Abstract

Pulmonary complications are known to occur in patients after Fontan palliation. Cardiac MRI is performed in the follow-up of Fontan patients to assess single ventricular function, hemodynamics and potential collateral flow. To date, pulmonary function tests have been used to detect functional lung impairment, but lung MRI has not been integrated into imaging follow-up. In this study, we measured lung-to-background ratio (LBR) on zero echo time (ZTE) MRI sequences in 32 children with Fontan palliation. Patients were divided into 2 groups: LBR > 1.5 and < 1.5 and assessed for associations between LBR and ventricular function, fibrosis, hemodynamics as well as biomarkers, spirometry and previous catheter results. We observed significantly increased extracellular volume (ECV) and decreased tissue inhibitor of metalloproteinase 1 (TIMP-1), soluble suppression of tumorigenicity 2 (sST-2) and a trend towards decreased growth differentiation factor 15 (GDF-15) as well as decreased albumin and prothrombin time values in patients with elevated LBR, whereas the other cardiovascular and pulmonary parameters did not differ significantly. Moreover, patients with LBR > 1.5 had a history of increased pulmonary arterial pressure prior to the Fontan and Glenn procedures and interventional veno-venous collateral embolization. Monitoring lung density with ZTE MRI sequences may be helpful in the diagnostic workup to assess the outcome of patients after Fontan procedure.

## Introduction

In patients with a single ventricle (SV) mid- and long-term survival after the Fontan operation is good. However, complications arising from their the non-physiological Fontan circulation cause increasing complications and pathophysiologic challenges during follow-up [1, 2].

Pulmonary complications are frequent in Fontan patients [3], especially restrictive ventilatory function, plastic bronchitis, and bronchial problems (e.g., stenosis, compression, malacia) [3–5]. Furthermore, altered development of the lung tissue secondary to abnormal pulmonary vascular development and chronic cyanosis has been hypothesized [6, 7]. In addition to residual shunts as a result of Fontan fenestration, several factors such as pulmonary arteriovenous malformations, veno-venous collaterals and chylothorax may contribute to hypoxia after Fontan surgery [8]. Some of these lung morbidities are associated with circulatory and pulmonary limitations and may contribute to Fontan deterioration and increased mortality [9].

Spirometry measurements and exercise capacity tests have been shown to be useful for assessing lung function in Fontan patients [3, 4, 9]. In addition, various biomarkers offer insights into cardiac function, myocardial remodeling, fibrosis, and eventually prognosis [10].

To the best of our knowledge, lung imaging as part of the cardiovascular follow-up in patients with Fontan circulation has not been routinely performed. In conventional MRI sequences, lung signal cannot be measured due to the lung parenchyma’s fast decay due to low proton density and susceptibility effects from air-tissue interface [11]. More recently, ultra-short echo time (UTE) and zero-echo-time (ZTE) sequences, which achieve echo times of almost zero, have been developed as promising techniques for lung imaging [12–17]. UTE MRI has been shown to provide a reliable assessment of lung parenchymal density compared to CT, avoiding exposure to ionizing radiation and serving as a promising alternative for Fontan patients requiring repeat imaging [18]. Lung density measurements may provide valuable information about the distribution of pulmonary blood flow. Changes in lung density may indicate alterations in pulmonary perfusion, transpulmonary gradient, or the presence of pulmonary pathology. Previous studies on different patients revealed an age-dependency of lung density with a rapid decrease in the first two years of life and constant values in childhood and adolescence expressed as a lung-to-background ratio (LBR) of 1.4 for ZTE imaging [16].

This study examines the relationship between an elevated LBR measured on ZTE images and key cardiovascular parameters in Fontan patients, including cardiac MRI findings, biomarkers of heart failure and fibrosis, pulmonary blood flow, hemodynamic measurements, and lung function parameters such as spirometry and exercise testing. Our purpose was to assess whether LBR provides additional insight into Fontan hemodynamics and clinical status.

## Methods

### Patient Population

This cross-sectional study included 32 children and adolescents with Fontan palliation who underwent cardiac MRI including lung-specific sequences and lung spirometry, all within a maximum interval of 4 months between both examinations (2019–2021). The clinical history and the invasive hemodynamic data obtained in the routine cardiac catheterization prior to the stage II and III of Fontan palliation were collected from the clinical information system.

Patients with incomplete imaging data (e.g., missing ZTE sequences or non-diagnostic image quality), significant motion artifacts affecting lung MRI analysis, or missing key laboratory/functional parameters (e.g., NT-proBNP, exercise test results) had been excluded a priori.

The study followed the ethical guidelines of the declaration of Helsinki for medical research involving human subjects. The study was approved by the local ethics authorities (KEK ZH: 2018-01878). Written informed consent was obtained from the children’s parents or legal guardians before both examinations.

### Magnetic Resonance Imaging

#### Lung MRI Sequences

All Fontan patients underwent cardiothoracic MRI examination as part of their clinical assessment including lung MRI using a ZTE sequence. The patients were scanned in supine position on a 1.5-Tesla system (first scanner: Discovery MR450, GE Healthcare, Waukesha, WI; second scanner: Signa Artist, GE Healthcare, Waukesha, WI). Due to a change of scanner during the study period, the patients were scanned with a respiratory motion-resolved four-dimensional ZTE (4D ZTE) sequence with retrospective soft gating on the first scanner, while the remaining patients were scanned with a three-dimensional zero echo-time (3D ZTE) sequence with prospective gating on the second scanner. The 4D ZTE sequence was not available on the second scanner. Both sequences were acquired in free breathing with respiratory gating using belts near the diaphragm to capture the physiological motion signal. The 4D ZTE protocol was selected to segment the whole respiratory cycle into 4 phases (4 bins) that were separately reconstructed [19]. Depending on the sharpness of the image, the phases corresponding to the end-expiration phase, i.e. the second or third phase, were used for the analysis. The 3D ZTE sequence acquired data during expiration / end expiration only.

The acquisition parameters for both sequences are shown in Table 1.

**Table 1:** Acquisition parameters of 3D ZTE and 4D ZTE

Parameter	3D ZTE	4D ZTE
TR (ms)	581	327
TE (ms)	0.02	0.02
No. of spokes segment	256	64
Flip angle (degrees)	2	2
FOV (mm2)	280 × 280	320 × 320
Slice thickness (mm)	2	1.5
In-plane resolution (mm)	1.0–1.4	1.0–1.4
Acquisition matrix	232 × 232	224 × 324
Receiver BW (kHz)	62.5	62.5
Scan time (range)	4–5 min	5 min 30 s—6 min 30 s

*TR* repetition time, *TE* echo-time, *FOV* Field of view, *BW* Bandwidth

### Lung-to-Background Ratio Analysis

The analysis of the lung signal intensity measurement was performed as previously described [17]. The parenchyma of both lungs was manually segmented by applying intensity thresholding, with careful attention to exclude high-intensity vessels. The average pixel signal values were then extracted. The open-source image analysis software Osirix MD version 11.03 (Pixmeo Sarl 2016, Bernex, Switzerland) was used for the lung segmentation.

**Fig. 1:**
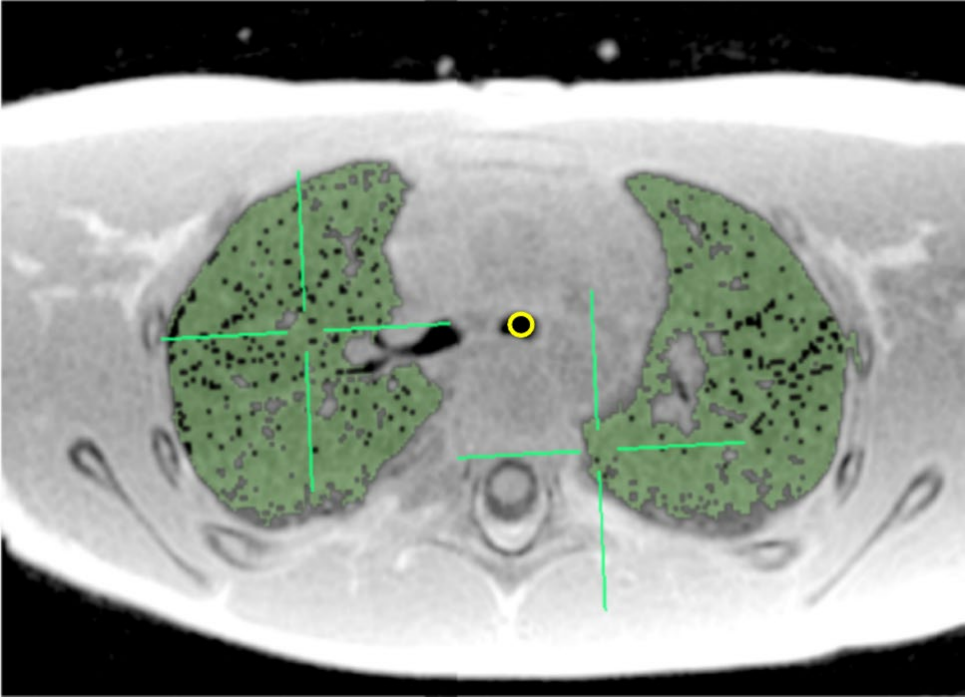
Manual lung segmentation by thresholding and region-of-interest placement in an 8-year-old female subject. Axial slice showing the manual segmentation of lung parenchyma (green) excluding high intensity vessels and with trachea (yellow) regions-of-interest

On the background air area, anterior to the patient’s chest, four two-dimensional regions-of-interest (ROI) (size of 20 mm^2^ each) were drawn and the average background signal intensity was measured. Another ROI of variable size was drawn inside the trachea on a single axial slice at the level of the carina to determine the signal intensity of the tracheal air column and thus validate that there were no artifacts inside the lung that could contaminate the measurement. Then, the lung-to-background ratio (LBR) was calculated as the ratio of the whole lung to trachea signal intensity (Fig. 1). As both sequences have different acquisition parameters and physical characteristics, the LBR normalizes the intensity to the respective noise level of each sequence, thus allowing comparison between sequences. The LBR threshold of 1.5 was selected based on previous research demonstrating that LBR values in healthy children and adolescents typically stabilize around 1.4 due to normal lung maturation [16, 17]. Values above 1.5 may therefore indicate early pathophysiological changes, allowing for a more precise differentiation between normal and abnormal pulmonary hemodynamics.

### Cardiac MRI

To measure ventricular size and function, steady-state free precession (SSFP) cine images were acquired in a short-axis plane covering the entire single ventricle using retrospective cardiac gating. Cardiac volumetry was assessed using semi-automated drawing of endocardial contours as previously described (QMass 4.0.62.4, MEDIS, Medical Imaging Systems, Leiden, The Netherlands) [20].

T1 mapping using a Modified Look-Locker Inversion Recovery (MOLLI) 3(3 s)3(3 s)5 sequence was acquired in a mid-ventricular short-axis slice with end-diastolic triggering and breath-hold [21]. T1 maps were created by segmenting the single ventricle pre and post-contrast enhancement and drawing a region of interest in the intracavitary blood space (Qmap T1, MEDIS). Extracellular volume (ECV) was calculated based on the formula:

$$ECV = (1/T1post - 1/T1pre)blood/(1/T1post - 1/T1pre)tissue \times (1 - Hematocrit)$$

As part of the standard clinical cardiac examination, a 4-point 4D flow acquisition was acquired in axial orientation covering the entire chest from the diaphragm to the aortic arch branches. The 4D flow sequence was acquired with electrocardiogram gating and free breathing, following a contrast-enhanced magnetic resonance angiography sequence. A k-space segmentation factor of three was used, and a k-t acceleration parallel imaging technique (*kat-ARC* = 8) was employed [22]. Blood flow in the systemic veins, pulmonary arteries and pulmonary veins was quantified using Arterys (Tempus Pixel, Chicago, Illinois, USA, 32.3.1). The cardiac MRI was concluded with late gadolinium enhancement sequences.

**Fig. 2:**
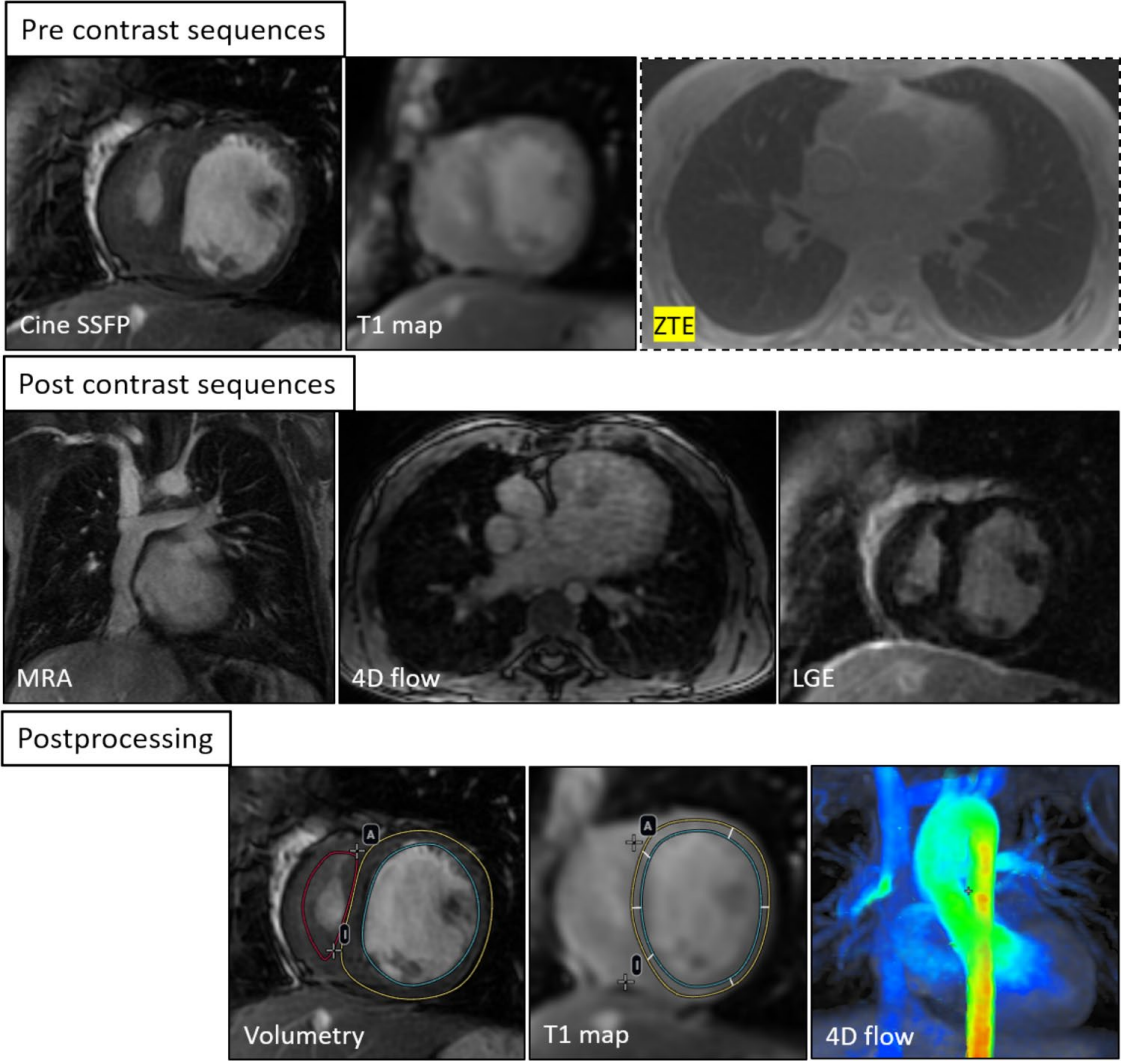
Workflow diagram summarizing the MRI acquisition protocol

The workflow diagram (Fig. 2) shows the order of the MRI sequences performed during the study.

### Spirometry/Lung Function Tests

Spirometry tests were performed in all patients according to the European Respiratory Society/American Respiratory Society guidelines using Masterlab (Jaeger, Würzburg, Germany). Main parameters included the expiratory volume in 1 s (FEV1), forced vital capacity (FVC), and the FEV1/FVC ratio. We calculated z-scores using the Global Lung Function Initiative reference Eqs. [23]. Results with z-score < −1.64 were defined as abnormal.

Cardiopulmonary exercise tests were performed on a cycle ergometer with a ramp protocol in accordance with our institutional standards [24]. Assessed parameters included maximal Oxygen Uptake (VO_2_max), Ventilation (VE), Ventilatory Equivalent for Oxygen (VE/VO_2_), Ventilatory Equivalent for Carbon Dioxide (VE/VCO_2_), Breathing Reserve (BR), Tidal Volume (VT), Breath Rate (BR), End-Tidal CO2 (PETCO2), and Oxygen Saturation (SpO2). Resting parameters were defined as the mean values of the raw data acquired during 3 min in a sitting position without cycling; the peak parameters were the mean values of the raw data acquired during the 30-s time interval at maximal exercise (peakVO2).

### Biomarkers

Heparin-plasma, and serum samples were collected, aliquoted into freezer vials, and stored at − 80 °C before analysis. All samples were only frozen and thawed once. Heparin-plasma was analyzed in an accredited diagnostic laboratory for growth differentiation factor 15 (GDF-15) on a Cobas e418 analyzer according to the manufacturer’s instructions using commercial reagents (Roche, Rotkreuz, Switzerland) with a coefficient of variation of 2.6% (7477 ng/l). Intact N-terminal propeptide of type III procollagen (PIIINP) and C-terminal telopeptide of type 1 collagen (CITP) were measured in serum with commercially available competitive radioimmunoassays (Orion Diagnostica Oy, Espoo, Finland) on a Wizard Gamma Counter 1470-002 (Perkin Elmer, Waltham, MA, USA) according to the manufacturer’s instructions with a coefficient of variation of 7.2% (12 µg/l) and 10% (4.3 µg/l), respectively. Preadipocyte factor 1 (DLK-1), tissue inhibitor of metalloproteinase 1 (TIMP-1), tissue inhibitor of metalloproteinase 4 (TIMP-4), and matrix metalloproteinase-2 (MMP-2) (TRayBio® ELISAs for Human Pref-1, TIMP-1, TIMP-4, and MMP-2, RayBiotech, Norcross, GA, USA) and soluble suppression of tumorigenicity 2 (sST2, CRITICAL DIAGNOSTICS, San Diego, CA, USA) were analyzed on a Synergy HT Multi-Detection Microplate Reader (BioTek, Winooski, VT, USA) according to the manufacturer’s instructions with a coefficient of variation of < 18% for the RayBiotech ELISAs and 8.5% (65.7 µg/l) for the CRITICAL DIAGNOSTICS ELISA, respectively.

Hepatic function was evaluated with albumin levels, prothrombin time, liver transaminases, fibrinogen, bilirubin, cholinesterase, and total protein. Patients on anticoagulation (*n* = 4) were excluded from the hepatic function analysis to avoid bias in coagulation-related markers such as Quick value. They were included in all other analyses, including LBR correlations with cardiac function, biomarker levels, and exercise capacity.

### Statistics

Statistical analysis was performed using SPSS 27.0.0 (SPSS Inc, IBM Company, Chicago Illinois, USA) and R (version 3.5.1; R Foundation for Statistical computing, Vienna, Austria). Continuous variables are expressed as median (interquartile ranges (IQR)), categorical data as counts (percentages). Kolmogorov–Smirnov analyses were used for group comparisons of non-normally distributed continuous variables. Ordinal, nominal, and dichotomous variables were evaluated with contingency tables and Chi-square-tests. Significant correlations were verified with logistic regressions and corrected for possible confounding factors (sex, age). Given our sample size (*n* = 32), we acknowledge the potential risk of overfitting and collinearity; however, we included this analysis as an exploratory approach to examine whether these variables retained significance after adjusting for confounders. Statistical significance was defined by values of *p* < 0.05.

## Results

### Patients

Thirty-two patients at a median age of 13.5 (11.1–13.5) years and weight of 45.8 (34.2–59.2) kg were included in the study. Primary diagnosis of a single right or single left ventricle were almost equally distributed, and hypoplastic left heart syndrome was the most frequent lesion (Table 2).

**Table 2:** Patient characteristics

Cardiac anatomy	*n*	%
Single left ventricle	17	53
Double inlet left ventricle	6	18
Tricuspid atresia	3	9
Pulmonary atresia with intact ventricular septum	6	18
Unbalanced atrioventricular septal defect	2	6
Single right ventricle	15	47
Hypoplastic left heart syndrome complex	11	37
Double outlet right ventricle	2	6
Unbalanced atrioventricular septal defect	2	6

Age at Fontan surgery was 2.5 (2.3–2.9) years, and the time interval between Fontan surgery and the study exams was 11.3 (8.3–13.9) years. There was no correlation between age at Fontan or time post-Fontan surgery and LBR. The Fontan operation consisted of an extracardiac tunnel in all subjects. At time of examination, transcutaneous oxygen saturation was 94 (92–97) % at rest, heart rate 86 (74–93) /min. Patients with LBR > 1.5 had a slightly earlier median Glenn age (5.3 months) compared to those with normal LBR (6.2 months), but this difference was not statistically significant.

Complications occurred in 10 (31%) patients and included history of chylothorax in five (15%), unilateral diaphragmatic palsy in three (9%), plastic bronchitis in two (6%), and protein-losing enteropathy in one patient (3%). Fenestration was closed in 93.8% (n = 30), open in 6.3% (*n* = 2). Chi-square analysis indicated no significant association between fenestration status and LBR.

A total of 24 (75%) of patients had an established therapy with beta-blockers, 6 (19%) had ACE-inhibitors, 4 (13%) had diuretics. Different medical therapies were not correlated with LBR.

Thirteen patients with a median age of 11.8 years (IQR: 10.1–14.3, male/female 8/5) were scanned using the 4D ZTE sequence. Nineteen patients were scanned with the 3D ZTE sequence (median age: 15.8 years, 12.3–16.6, male/female 8/11). There was no significant age difference between the two groups (*p* = 0.30).

### Lung-to-Background Ratio

The median LBR was 1.34 (1.32–1.41) for the 4D ZTE sequence and 1.34 (1.28–1.41) for the 3D ZTE sequence (*p* = 0.4). No statistically significant difference was observed between the right and left lung (p > 0.05; linear regression slope 0.98 with a very strong square coefficient of correlation R^2^ 0.99).

**Fig. 3:**
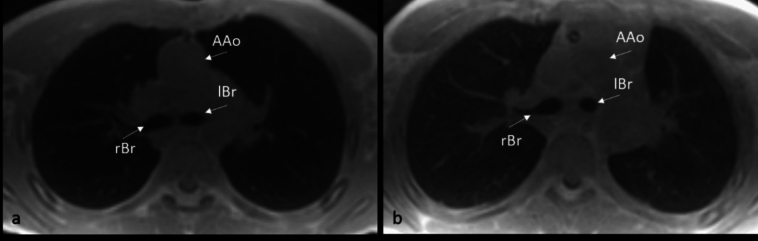
Representative images of 4D ZTE in axial orientation at the level of the carina in an 8-year-old male patient with normal LBR of 1.4 (**a**) and in a 10-year-old female patient with elevated LBR (1.6) (**b**). AAo ascending aorta, rBr right main bronchus, lBr left main bronchus

**Fig. 4:**
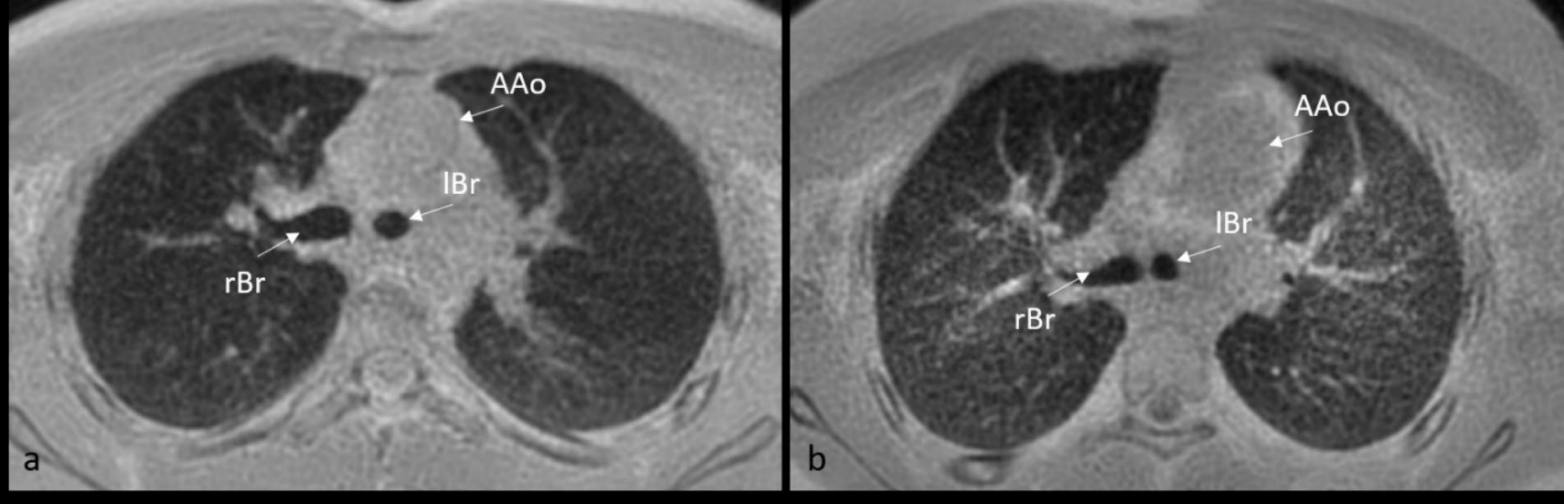
Representative images of 3D ZTE in axial orientation at the level of the carina in a 14-year-old male patient with normal LBR of 1.3 (**a**) and in a 12-year-old female patient with elevated LBR (1.7) (**b**) AAo ascending aorta, rBr right main bronchus, lBr left main bronchus.

Twenty-six patients had LBR values within the normal range of 1.3–1.4. Six patients had an elevated LBR > 1.5, with a maximum LBR of 1.75. Two of them were scanned with the 4D ZTE sequence and four with the 3D ZTE sequence. Figures 3 and 4 show exemplary cases with normal and pathological LBR scanned with the 4D ZTE and the 3D ZTE sequence, respectively.

Patients with normal and high LBR did not differ with respect to age, body mass index, heart rate, age at Fontan palliation or years after Fontan surgery. A systemic right ventricle was present in 5/6 patients with an elevated LBR (p = 0.04). No difference was observed regarding the incidence of complications such as chylothorax or diaphragmatic palsy.

### Cardiac Magnetic Resonance Analysis

**Fig. 5:**
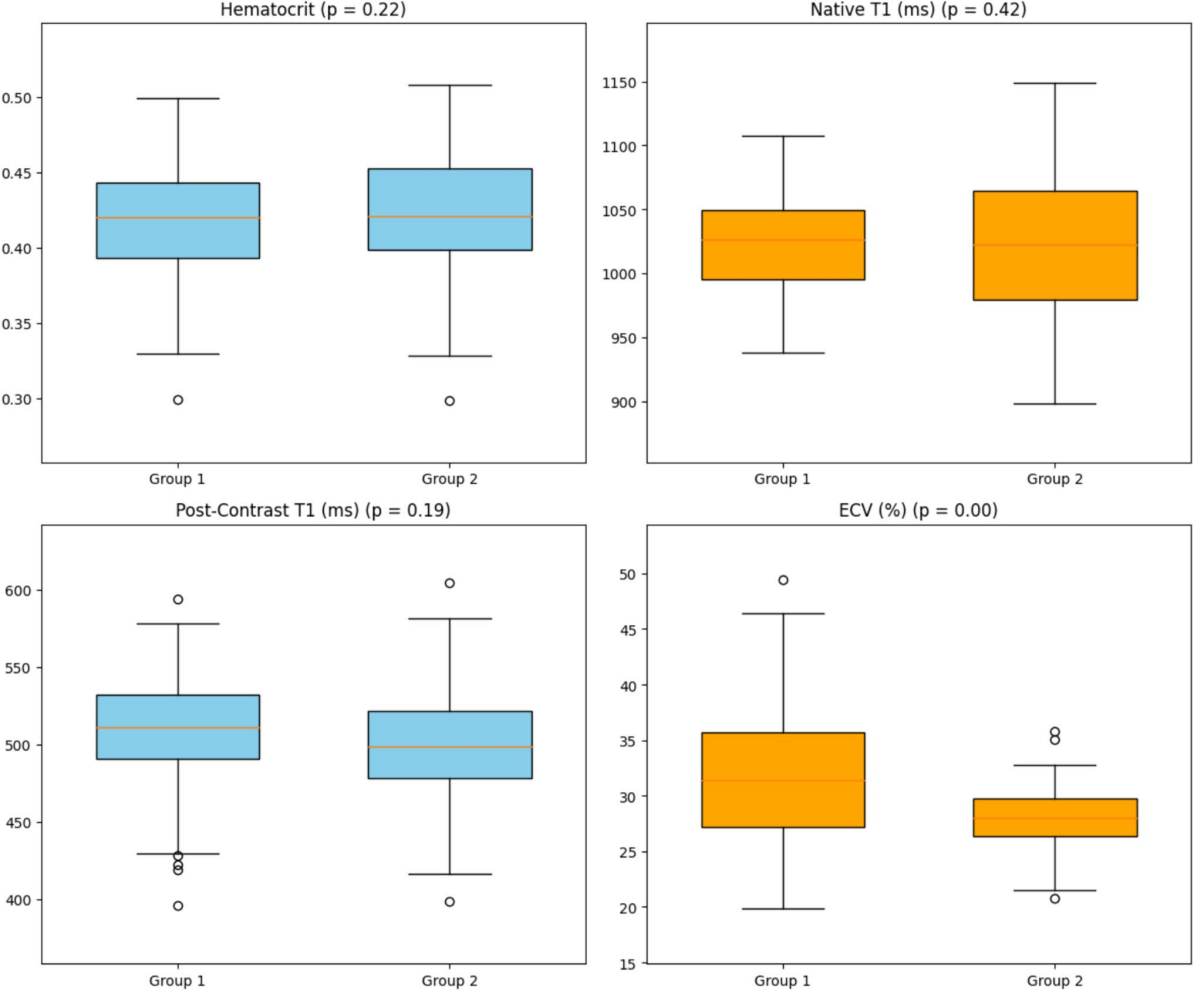
Box plots highlighting the significant difference in extracellular volume (ECV) (*p* < 0.0001) and the similarity of the other T1-mapping parameters and hematocrit used to calculate ECV between the patients with elevated LBR measurements (group 1) and the other patients (group 2)

Cardiac MRI parameters are summarized in Table 3 and Fig. 5. ECV values were significantly higher in patients with increased LBR compared to those with normal LBR (30% vs. 27%; *p* < 0.001). In contrast, no difference was found for native and post-contrast myocardial T1 mapping values, single ventricle volume and function, and pulmonary flow distribution.

**Table 3:** Cardiac magnetic resonance analysis

	High LBR	Normal LBR	*p*
ESV (ml/m^2^)	47.0 (43.5–58.7)	52.0 (35.5–73.7)	0.91
EDV (ml/m^2^)	125.0 (96.2–153.0)	116.0 (88.0–139.7)	0.86
Ejection fraction (%)	53.5 (48.9–63.0)	50.0 (47.7–58.6)	0.49
RPA/LPA ratio	1.23 (1.06–1.66)	1.55 (1.32–1.9)	0.12
**ECV (%)**	**30%; 27–38**	**27%; 25.7–28.9**	** < 0.001**
T1 mapping native (ms)	1020 (983–1050)	1021 (975–1048)	0.42
T1 mapping post contrast (ms)	492 (480–545)	500.00 (469–537)	0.19
Hematocrit	0.40 (0.40–0.45)	0.41 (0.40–0.43)	0.22

Bold values indicate the statistically significant results

*EDV* end-diastolic volume, *ESV* end-systolic volume, *ECV* Extracellular volume, *LBR* lung-background-ratio

### Invasive Hemodynamic Evaluation

Invasive hemodynamic data from the routine cardiac catheterizations performed before Glenn and Fontan procedure were assessed. Patients with increased LBR values had higher mean pulmonary arterial pressure (mPAP) prior to the Glenn surgery (median of 15 (13–18) mmHg versus a median of 12 (11–14) mmHg (*p* = 0.04)), and prior to the Fontan surgery (15 (12–17) versus 12 (11–13) mmHg (*p* = 0.049)). The end-diastolic ventricular pressure was not significantly different. Patients with elevated LBR more often underwent interventional closure of veno-venous collaterals (*p* = 0.04).

### Spirometry and Exercise Capacity Results

LBR values did not correlate with the results of functional lung tests at rest (spirometry) and under exercise (cardiopulmonary exercise test). No significant correlation was found between LBR and pulmonary function parameters, including spirometry (FVC, FEV₁), peak oxygen uptake (VO₂ peak), breathing frequency, and oxygen uptake efficiency slope (OUES).

### Hepatic Function

Patients with elevated LBR values had lower albumin levels (42 g/L (39–44) versus 45 g/L (43–47), *p* = 0.043) and lower prothrombin time (Quick) (68% (57–75) versus 83% (78–88), *p* = 0.049). Liver transaminases, fibrinogen, bilirubin, cholinesterase and total protein were similar between the groups.

### Biomarkers

**Fig. 6:**
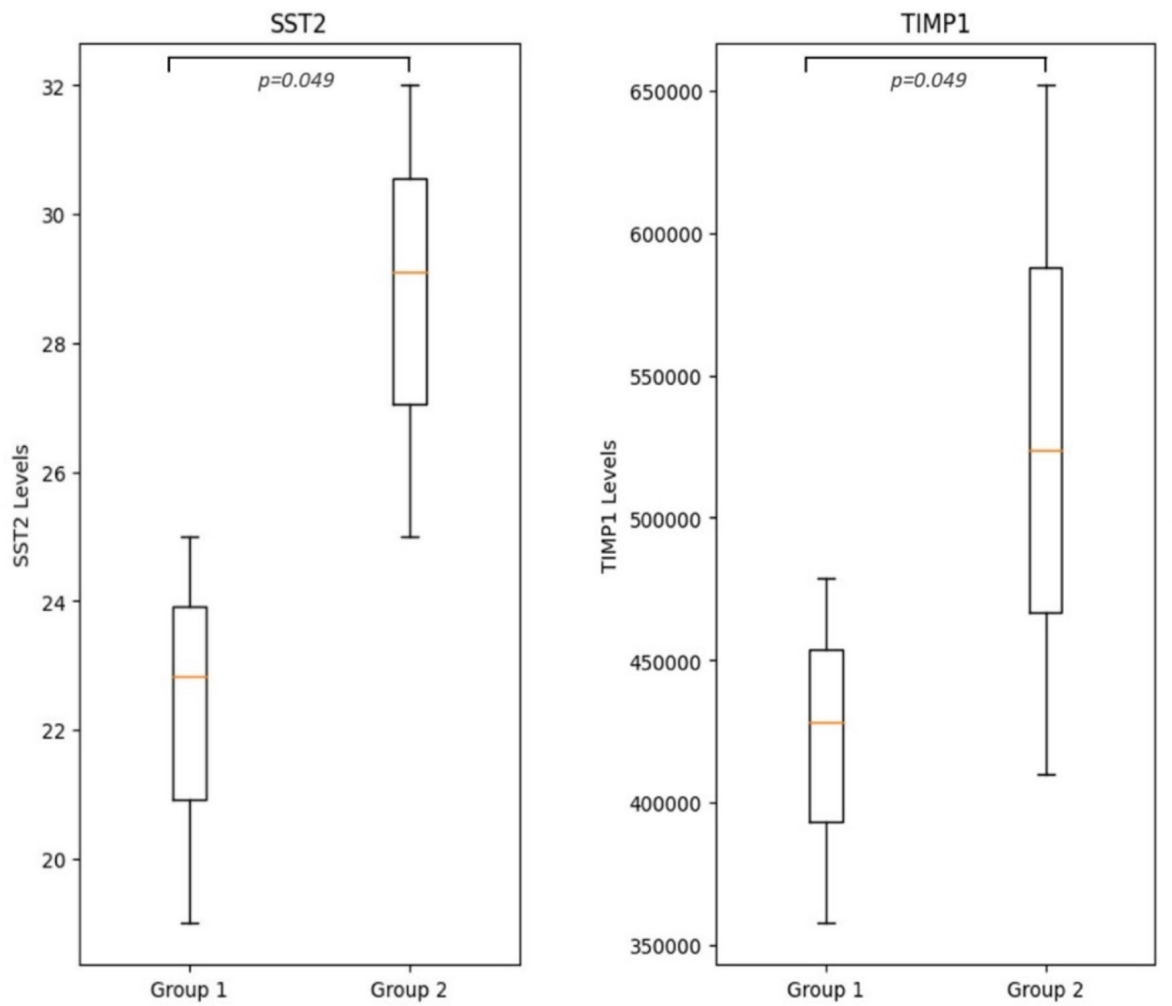
Box plots showing the lower level of TIMP1 (pg/mL) and sST2 (ng/mL) in the patients with elevated LBR measurements (group 1) versus the patients with normal LBR (group 2)

Biomarker analysis revealed significant differences between patients with elevated LBR (> 1.5) and those with normal LBR (≤ 1.5). Specifically, patients with higher LBR exhibited lower levels of biomarkers associated with myocardial remodeling and fibrosis. TIMP-1 levels were significantly reduced in the high LBR group (428 pg/mL [358–479] vs. 524 pg/mL [410–652], *p* = 0.049), as were sST-2 (22 ng/mL [19–25] vs. 29 ng/mL [25–32], *p* = 0.049) and GDF-15 (479 pg/mL [420–520] vs. 601 pg/mL [530–690], *p* = 0.056) (Fig. 6).

In contrast, NT-proBNP levels did not differ significantly between the groups.

### Independent Factors Associated with Elevated LBR

On multiple regression (*χ*^2^ = 18.908, *p* = 0.003, Nagelkerke *R*^2^ = 0.749) with ECV (OR = 13.88, 95% CI: 3.50–55.00, *p* < 0.001), mPAP prior to Glenn (OR = 2.56, 95% CI: 1.01–6.50, *p* = 0.048), TIMP-1 (OR = 0.27, 95% CI: 0.07–0.99, *p* = 0.049), SST-2 (OR = 0.29, 95% CI: 0.09–0.98, *p* = 0.043) showed an independent relation to LBR corrected for age, years from Fontan, sex, and weight.

## Discussion

In this study, we have measured lung density by using ZTE MRI sequences in a cohort of pediatric Fontan patients. Our hypothesis was that alterations in pulmonary perfusion, cardiac or lung function parameters may change lung density values. Our results show that an increased lung density, expressed as a LBR > 1.5, is observed in patients with an increased myocardial ECV and with abnormal serum biomarkers, including decreased TIMP1, sST2-, albumin and prothrombin time values. In contrast, other functional parameters, such as ventricular volume, function, myocardial T1 values, pulmonary blood flow on MRI and lung function measures by spirometry did not correlate with LBR.

In recent years, the UTE and ZTE MR sequences have been increasingly impacting the role of MRI in lung imaging, with replacing CT, which has been the gold standard in clinical routine for decades [13–15, 25, 26]. The use of radiation-free imaging modalities is in demand, particularly in children, as they are more sensitive to radiation [27]. Various types of UTE and ZTE sequences have been developed and investigated for their clinical applicability, e.g. in the context of cystic fibrosis, lung nodule detection or lung parenchyma characterization [12, 13, 15, 19]. The use of these sequences for measuring lung density has been reported in previous studies, which described the age-dependent values of LBR and reported a normal LBR value of 1.3–1.4 after two years of age [16]. Based on this normative value, we defined a LBR > 1.5 as pathological and could identify six patients with increased LBR values for further analysis.

Asymmetric pulmonary perfusion measured by 4D Flow MRI does not seem to represent a cause of the elevated LBR values. As we did not observe correlations between increased LBR values and lung function (both at rest and during exercise), we believe that a structural parenchymal lung pathology does not reflect the altered LBR values. This could indicate that structural lung changes do not necessarily impair ventilatory efficiency or that alternative compensatory mechanisms maintain functional capacity.

Interestingly, patients with elevated LBR exhibited significantly higher mean pulmonary artery pressure prior to the Glenn and Fontan procedure, but no significant difference in end-diastolic pressure, resulting in an increased transpulmonary gradient, and the same patients underwent more frequently interventional veno-venous collateral occlusion. The elevated pulmonary pressure values and more frequent venous collateral closures during catheterization [28], suggest a potentially impaired pulmonary circulation in these patients.

Patients with a systemic right ventricle typically face a greater long-term hemodynamic burden due to the right ventricle's inherent inefficiency in sustaining systemic circulation. This physiological disadvantage may contribute to the higher LBR values observed in these patients.

The observed lower albumin and prothrombin time values may be related to the increased Fontan pressure in the patients with pathological LBR > 1.5.

In terms of cardiac volumetry, function and myocardial characteristics, we found a significant difference in ECV, a marker for quantifying extracellular volume. This was particularly interesting as native and post-contrast T1 mapping values and hematocrit did not differ between the two groups. This suggests that patients with higher LBR values also have a greater degree of myocardial extracellular matrix expansion, which is likely to be indicative of fibrosis.

Among the various biomarkers analyzed, sST-2, TIMP-1, and GDF-15 were found to be reduced in patients with elevated LBR values. sST-2 is a marker associated with myocardial stretch and fibrosis, TIMPs regulate extracellular matrix remodeling, and GDF-15 is linked to inflammation and oxidative stress.

In contrast to prior studies that associated higher levels of these biomarkers with worse clinical outcomes, our findings suggest that Fontan patients with higher LBR exhibit lower levels of TIMP-1, sST-2, and GDF-15. For example, elevated sST-2 and GDF-15 levels were previously identified as predictors of adverse events in Fontan patients [10]. Similarly, Raedle-Hurst et al. [29] demonstrated that GDF-15 correlated with reduced single ventricle ejection fraction, while Meyer *et al*. [30] linked higher GDF-15 levels to increased mortality and rehospitalization rates. However, a direct comparison with our study is challenging, as both Raedle-Hurst et al. and Meyer et al. reported higher absolute GDF-15 levels than those observed in our cohort. Additionally, van den Bosch et al. [10] reported only correlations between biomarker levels and outcomes, without specifying the absolute biomarker values, limiting direct comparison.

Interestingly, Nakano et al. [31] found lower myocardial TIMP-1 levels in failing Fontan patients, suggesting a possible downregulation of TIMP-1 in progressive Fontan failure, which aligns with our findings of lower TIMP-1 in patients with elevated LBR. TIMP-1 reduction has also been reported in adults with dilated cardiomyopathy [32] reinforcing the hypothesis that altered matrix remodeling could contribute to these observations.

Despite these findings, normative biomarker values in Fontan patients remain undefined, making interpretation difficult. The observed results may reflect heterogeneous phenotypes within the Fontan population or may indicate dynamic processes that are not fully captured in a cross-sectional analysis. For example, lower GDF-15 levels may indicate a reduced ability to mount a stress response, whereas lower sST-2 and TIMP-1 levels might reflect attenuated extracellular matrix remodeling. The absence of significant differences in other fibrosis-related biomarkers suggests that elevated LBR may be associated with selective pathway alterations rather than widespread fibrotic remodeling.

The main limitation of our study was the change of the scanner during the ongoing study, with a simultaneous change in the type of ZTE sequence from 4D ZTE to 3D ZTE. It is known that signal and noise around structures with very short T2*, such as the surrounding coils, can propagate into the background noise outside the chest. To avoid such errors in the LBR measurement, we used the intraluminal air of the trachea instead of the extra-corporeal air as the background reference. We acknowledge that our sample size (*n* = 32) is relatively small, potentially limiting statistical power and generalizability. The limited size of our cohort and the cross-sectional design of the study complicate the interpretation of the results, which must be considered as suggestive. Data on these quite novel parameters from a larger group of patients and in a more dynamic prospective during follow-up may help to clarify the interdependence of pathophysiological changes in different organ systems and to better define which non-invasive parameters may be useful for risk stratification. A further limitation is that all MRI examinations were performed at rest. Exercise stress imaging protocols could further elucidate whether post-exercise changes in lung density contribute to Fontan hemodynamics. Future research should address this issue.

In conclusion, our preliminary results indicate that increased lung density with higher LBR values may be associated with a less efficient Fontan circulation with higher pulmonary pressures. This seems to be supported by the altered liver function and myocardial structure. Moreover, lung density measurements may be more sensitive than other functional analyses and may describe subclinical changes, as we didn’t find any differences in rest and exercise lung function in patients with increased LBR. Prospective follow-up studies monitoring the values of lung density over time are needed to test this hypothesis.

## Data Availability

The authors confirm that the data supporting the findings of this study are available within the article. Supplementary data that support the findings of this study are available from the corresponding author, AC, upon reasonable request.
